# Synthetic short-chain peptide analogues of H1 relaxin lack affinity for the RXFP1 receptor and relaxin-like bioactivity. Clues to a better understanding of relaxin agonist design

**DOI:** 10.3389/fphar.2022.942178

**Published:** 2022-08-11

**Authors:** Annunziata D'Ercole, Silvia Nistri, Lorenzo Pacini, Alfonso Carotenuto, Federica Santoro, Anna Maria Papini, Ross A. D. Bathgate, Daniele Bani, Paolo Rovero

**Affiliations:** ^1^ Interdepartmental Research Unit of Peptide and Protein Chemistry and Biology, University of Florence, Florence, Italy; ^2^ Department of Chemistry “Ugo Schiff”, University of Florence, Florence, Italy; ^3^ Research Unit of Histology & Embryology, Department of Experimental & Clinical Medicine, University of Florence, Florence, Italy; ^4^ Department of Pharmacy, University of Naples Federico II, Naples, Italy; ^5^ Florey Institute of Neuroscience and Mental Health and Department of Biochemistry and Pharmacology, Unviversity of Melbourne, Melbourne, VIC, Australia; ^6^ Department of NeuroFarBa, University of Florence, Florence, Italy

**Keywords:** relaxin, RXFP1, relaxin analogues, cAMP, ERK1/2, RXFP1 agonists

## Abstract

The peptide hormone relaxin (RLX), also available as clinical-grade recombinant protein (serelaxin), holds great promise as a cardiovascular and anti-fibrotic agent but is limited by the pharmacokinetic issues common to all peptide drugs. In this study, by a computational modelling chemistry approach, we have synthesized and tested a set of low molecular weight peptides based on the putative receptor-binding domain of the B chain of human H1 RLX isoform, with the objective to obtain RLX analogues with improved pharmacokinetic features. Some of them were stabilized to induce the appropriate 3-D conformation by intra-chain tri-azolic staples, which should theoretically enhance their resistance to digestive enzymes making them suited for oral administration. Despite these favourable premises, none of these H1 peptides, either linear or stapled, revealed a sufficient affinity to the specific RLX receptor RXFP1. Moreover, none of them was endowed with any RLX-like biological effects in RXFP1-expressing THP-1 human monocytic cells and mouse NIH-3T3-derived myofibroblasts in *in vitro* culture, in terms of significantly relevant cAMP elevation and ERK1/2 phosphorylation, which represent two major signal transduction events downstream RXFP1 activation. This was at variance with authentic serelaxin, which induced a clear-cut, significant activation of both these classical RLX signaling pathways. Albeit negative, the results of this study offer additional information about the structural requirements that new peptide therapeutics shall possess to effectively behave as RXFP1 agonists and RLX analogues.

## Introduction

Since recombinant human H2 relaxin (RLX), later termed serelaxin, was made available as clinical-grade drug (Stults et al., 1990), several clinical trials have been performed to explore its therapeutic potential, particularly as cardiovascular and anti-fibrotic agent (Bani 2020; Samuel and Bennett 2021; Sassoli et al., 2022). The last RELAX-AHF-2 phase III trial was performed under the auspices of Novartis Europharm Ltd., owner of the serelaxin patent, to assess its efficacy in patients with heart failure. Regrettably, in spite of encouraging theoretical premises and pre-clinical evidence, the results of these studies failed to demonstrate a substantial improvement of the main symptoms and signs and of life expectancy in the RLX-treated vs. placebo-treated patients (Teerlink et al., 2013), thus inducing Novartis to give up this line of investigation. A careful reappraisal of the possible reasons behind this disappointing outcome has identified some critical issues, among which a major place is held by the short half-life (∼2 h) of serelaxin upon i.v. administration (Chen et al., 1993), forcing it to be given parenterally in multiple daily doses, with obvious drawbacks in terms of ease of use, patients’ compliance and suitability for long-term treatments (Bani 2020). These limitations of serelaxin, shared by many peptide drugs, have prompted the pharmaceutical research towards the synthesis of low molecular weight serelaxin analogues, designed to retain the desired biological effects of the authentic peptide with the advantages of improved resistance to proteolytic catabolism, extended half-life and suitability for oral delivery (Praveen et al., 2019). To behave as *bona fide* serelaxin analogues, such molecules should be able to bind to and activate the specific RLX receptor RXFP1 (Park et al., 2008; Bathgate et al., 2013). This is complicated by the peculiarly complex ligand-receptor interaction mode (Sethi et al., 2021). In fact, the natural hormone is composed of 2 peptide chains, A and B, stabilized by intra-and inter-chain disulfide bonds, of which the A chain basically has a stabilizing function pivotal for receptor affinity, while the B chain bears the receptor-binding domain, made up by an arginine cassette including Arg13, Arg17 and Ile20, located on the same face of an α-helix, typical of the hormone bioactive conformation (Bathgate et al., 2013). On the other hand, RXFP1, a G-protein-coupled receptor (GPCR), has a complex structure which encompasses a classical 7-loop trans-membrane domain (TMD), a large extracellular domain of leucine-rich repeats (LRR) which is joined to a N-terminal low-density lipoprotein-a (LDLa) domain via a short linker region (Bathgate et al., 2013). RLX binds with high affinity to the LRRs and low affinity to the linker enabling the LDLa-linker domain to form a conformation leading to G-protein-dependent signal transduction pathways, which differ depending on the target cell types (Halls et al., 2007; Sethi et al., 2021). The most common signaling mechanisms downstream of RXFP1 include the elevation of cAMP, the increased phosphorylation of extracellular-regulated protein kinases (ERK) 1/2 (Hossain et al., 2016) and the up-regulation of nitric oxide production (Baccari & Bani 2008). This unique mode of interaction of RLX with its receptor has represented a challenge for the generation of effective RXFP1 agonists. To date, three different conceptual pathways have been explored, namely: peptide and non-peptide RXFP1 agonists designed on receptor complementarity (Shemesh et al., 2009; Xiao et al., 2013; Agoulnik et al., 2017), low molecular weight RLX analogues designed on the functional B chain domains (Hossain et al., 2016; Marshall et al., 2017; Mallart et al., 2021) and semi-synthetic double-chain RLX analogues modified to extend their half-life (Muppidi et al., 2019). Another approach focuses on H1 RLX, whose gene *Rln1* is only present in primates as a likely ortholog of the RLX-encoding gene *Rln2* (Hansell et al., 1991). Recombinant H1 RLX was found to have comparable RXFP1 affinity as H2 RLX (Bathgate et al., 2006), as well as similar chronotropic and inotropic effects in the isolated rat heart assay (Wade et al., 1996), Moreover, H1 RLX showed a greater alpha-helical conformational preference in water than H2 RLX and retained cardiotropic effects even upon modification of the C-terminus of the B-chain (Wade et al., 1996). On these grounds, we reasoned that low molecular weight, single-chain synthetic analogues of H1 RLX would deserve to be investigated as possible RXFP1 agonists. The underlying rationale was based on the following points: 1) shorter peptide, bearing unnatural modifications, such as triazole bridges, are potentially endowed with greater enzymatic stability and thus are better drug candidates; 2) the RLX B chain, characterized by the presence of the receptor binding domain, is a good template for the design of receptor subtype selective analogues. In this context, selective analogues of several peptide hormones have been developed using fragments or partial sequences as starting point (Maggi et al., 1990). In general, while several synthetic and recombinant analogues of H2 RLX have been extensively investigated, the study of the structure-activity relationships of H1 RLX and the development of either synthetic or recombinant analogues has been much less advanced.

In the present study, we have designed, synthesized and tested *in vitro* a set of low molecular weight peptides based on the putative receptor-binding domain of the H1 RLX B chain with the aim of identifying a novel class of RXFP1 agonists potentially exploitable as RLX-mimetic drugs.

## Materials and methods

*Materials*—Serelaxin (batch B917056/1/1, prepared by Boehringer-Ingelheim Inc.) was kindly donated by the Relaxin RRCA Foundation, Florence, Italy. Short-chain peptide analogues of H1 RLX (Table 1) and the known RXFP1 agonist B7-33 (Hossain et al., 2016) were synthesized as previously described (D'Ercole et al., 2020; Nuti et al., 2010). Full analytical characterization (HPLC and MS) of the synthetic peptides is available as Supplementary Material (Table 1). Stock aliquots of each substance (80 μM) were stored at −80°C and thawed immediately before further use. Silicon-coated test tubes were used to prevent adhesion of the peptides to the walls.

**TABLE 1:** H1 RLX peptide analogues.

No.	Name	Structure*
**1**	[Ser^10,22^]H1RLXB(1–23)	K-W-K-D-D-V-I-K-L-S-G-R-E-L-V-R-A-Q-I-A-I-S-G
**2**	[Ser^22^]Cyclo[Pra^10^, LysN_3_^14^]H1RLXB(1–23)	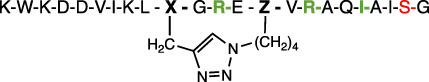
**3**	[Ser^22^]Cyclo[LysN_3_^10^, Pra^14^]H1RLXB(1–23)	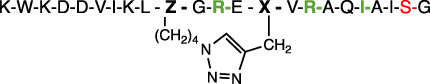
**4**	[Ser^10,22^]Cyclo[Pra^14^, LysN_3_^18^]H1RLXB(1–23)	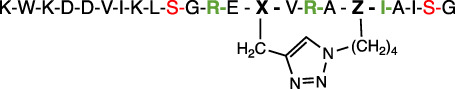
**5**	[Ser^10,22^]Cyclo[LysN_3_^14^, Pra^18^]H1RLXB(1–23)	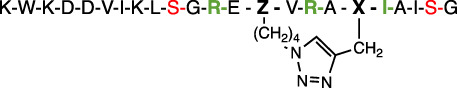
**6**	[Ser^10,22^]Cyclo[Pra^17^, LysN_3_^21^]H1RLXB(1–23)	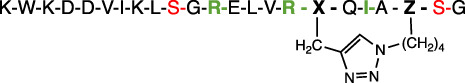
**7**	[Ser^10,22^]Cyclo[LysN_3_^17^, Pra^21^]H1RLXB(1–23)	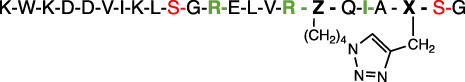

^∗^ **X**, Pra; **Z**, LysN_3_.

Unless otherwise stated, all chemicals and reagents used in the experiments were from Sigma-Aldrich (Milan, Italy), while cell culture plastic ware was from VWR-Avantor (Milan, Italy).

*Cell culture for ligand-receptor binding assays*—Human embryonic kidney cells (HEK-293T) stably expressing RXFP1-BP (Halls et al., 2005) were used for competition binding experiments using europium-labelled H2 relaxin, as described (Shabanpoor et al., 2012). This construct contains the ectodomain of human RXFP1 fused to the single transmembrane and cytoplasmic region of CD8. This ectodomain-only construct has a higher affinity for single chain H2 relaxin peptides and is used as a surrogate assay for binding affinity. Assays were performed in whole cell 96 well plates assays with a single concentration of the europium-labeled H2 relaxin (1 nm) in the presence or absence of increasing concentrations of the competing peptides. Non-specific binding was assessed using 1 µM of serelaxin. Each concentration point was assessed in triplicate in 2 independent experiments. The binding data were analyzed using GraphPad Prism 9 and expressed as mean ± SEM. They were fitted using a one site binding model.

*Cell culture for cAMP assay*—The RXFP1 downstream cAMP pathway was tested on human monocytic THP-1 cells (ECACC, Salisbury, UK) cultured in suspension in RPMI medium containing 10% foetal bovine serum, 0.05 mm 2-mercaptoethanol, 250 U/ml penicillin G and 250 μg/ml streptomycin, in a 5% CO_2_ atmosphere at 37°C. Cells (5 × 10^5^) were placed in a 24-well plate, added with IBMX (100 μM) to prevent cAMP catabolism and then incubated with the different stimulants. As positive controls, either serelaxin (17 nm) and B7-33 (17, 170, 1700 nm) were used. The H1 peptides **4**, **6** and **7**, selected among those which had shown a minimal ability to bind RXFP1 in the competition binding experiments, were also used at 17, 170 and 1700 nm final concentration. The cAMP assay was carried out 15 min after RXFP1 stimulation, coinciding with the second sustained cAMP surge (Nguyen et al., 2003; Bani et al., 2007). The adenylate cyclase activator forskolin (100 μM) was used to determine the maximal cAMP levels. Triplicate cAMP measurements were performed by a Direct cAMP ELISA Kit (Enzo, Milan, Italy). Results were calculated using a 4 parameter logistic (4PL) curve fitting program, as suggested by the manufacturer. The values of cAMP were normalized by the amount of proteins, measured by the micro-BCA Protein Assay Kit (Pierce, IL, United States) and expressed as mg/ml. For statistical purposes, 3 independent experiments were performed.

*Cell culture for ERK1/2 phosphorylation assay* - The RXFP1 downstream ERK1/2 phosphorylation pathway was tested on myofibroblasts using a method based on that described by Hossain et al. (2016), with minor modifications. Mouse NIH-3T3 fibroblasts (ATCC, Manassas, VA, United States) expressing RXFP1 (Sassoli et al., 2013) were cultured in DMEM supplemented with 10% fetal bovine serum (FBS), 2 mm glutamine, 250 U/ml penicillin G and 250 μg/ml streptomycin, in a humidified atmosphere with 5% CO_2_ at 37°C. They were induced to myofibroblasts in DMEM containing 2% FBS and 2 ng/ml human TGF-β1 (PeproTech, Rocky Hill, NJ, United States) for 12, 24, 48 and 72 h (Sassoli et al., 2013). Myofibroblasts were then incubated for 30 or 90 min with either serelaxin (17 nM), or B7-33 (1.7 μM), or the H1 peptides **6** and **7** (1.7 μM), selected among those which had shown a minimal ability to bind RXFP1 in the competition binding experiments. Untreated myofibroblasts were used as controls. To verify fibroblast-to-myofibroblast transition and ERK1/2 phosphorylation, cells were lysed in cold buffer composed of (mM): 10 Tris/HCl pH 7.4, 10 NaCl, 1.5 MgCl_2_, 2 Na_2_EDTA, 1% Triton X-100, added with 10x Sigmafast Protease Inhibitor Cocktail tablets and 100 mM Na_3_VO_4_ to inhibit endogenous phosphatases. Total protein content was measured spectrophotometrically using micro-BCA Protein Assay Kit (Pierce). Sixty μg of total proteins were electrophoresed by SDS–PAGE and blotted onto PVDF membranes (Millipore, Bedford, MA, United States). The membranes were incubated overnight at 4°C with: goat polyclonal anti-α-smooth muscle actin (α-sma, 1:1,500, AbCam, Cambridge, UK); rabbit polyclonal anti-pERK1/2 (1:1,000, Cell Signaling, Milan, Italy), rabbit polyclonal anti-ERK1/2 (1: 1,000, Cell Signaling), mouse monoclonal anti-GAPDH (1:2000; Invitrogen, Waltham, MA, United States), assuming GAPDH as control invariant protein. Specific bands were detected using appropriate peroxidase-labeled secondary antibodies (1:15,000; Vector, Burlingame, CA, United States) and enhanced chemiluminescent substrate (ECL, Sigma-Aldrich). Densitometric analysis of the bands was performed using Scion Image Beta 4.0.2 software (Scion Corp., Frederick, MD, United States) and the values normalized to GAPDH. For statistical purposes, 3 independent experiments were performed.

## Results

### Peptide analogues design and synthesis

We focused our attention on the B chain of H1 RLX, with the aim of stabilizing the putative bioactive conformation of the LRR-binding domain through the introduction of a conformational constraint based on side chain-to-side chain, i to i + 4 triazole bridge, obtained by copper (I)-catalyzed alkyne-azide cycloaddition (CuAAC) (Testa et al., 2018). As a proof of principle, we recently published the synthesis of a couple of such H1 RLX stapled analogues (D'Ercole et al., 2020). Building on these preliminary data, we describe here a series of stapled peptide analogues of H1 RLX B chain, prepared using an optimized microwave-assisted synthetic strategy (Rizzolo et al., 2011). In the absence of specific structure-activity relationship studies, we decided to use as a starting point the C-terminal truncated fragment of H1 RLX B chain H1RLX(1–23) (peptide **1** in Table 1), which is expected to adopt an α-helical conformation in the native hormone, on the basis of the structural data available for H2 RLX (Hossain et al., 2011). We replaced Cys residues in positions 10 and 22 by Ser, to avoid unwanted formation of disulphide bridges and we installed the α-helix inducing i to i + 4 triazole bridge modifying, one at a time, three couples of positions not directly involved in receptor binding, i.e., 10-14, 14–18, and 17–21, respectively. Accordingly, as shown in Table 1, the conformational constraint in position 10–14 was obtained replacing Ser10 by Pra and Leu14 by Lys (N3), or, vice versa, Ser10 by Lys (N_3_) and Leu14 by Pra, obtaining, after CuAAC reaction, the stapled cyclo-peptides **2** and **3**, respectively, characterized by opposite orientation of the triazole moiety, as shown in Table 1. Similarly, peptides **4** and **5** bear the triazole bridge in position 14–18 and peptides **6** and **7** in position 17–21, respectively.

### Conformational studies (circular dichroism)

**FIGURE 1:**
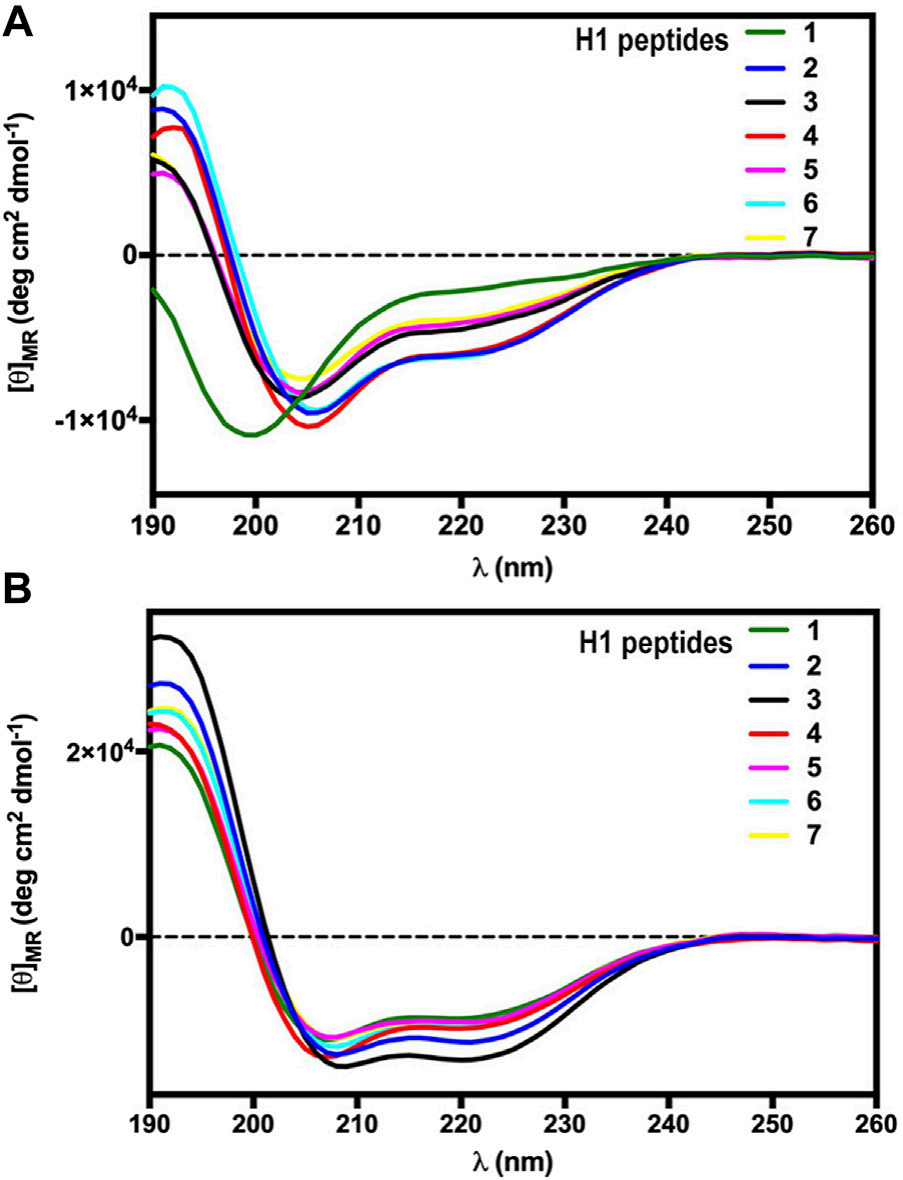
CD spectra of the H1 RLX stapled analogues (**2**–**7**) and the linear reference peptide **1** in phosphate buffer **(A)** and in SDS micelles **(B)**.

**FIGURE 2:**
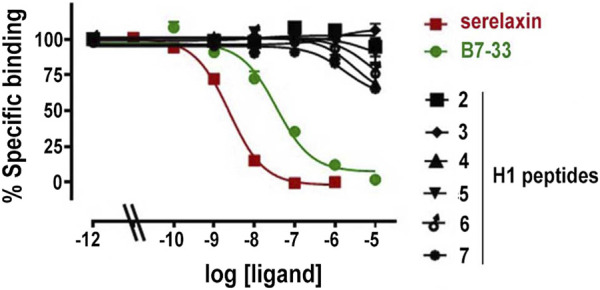
Competition binding curves for serelaxin, B7-33 and H1 peptides in HEK-293T stably expressing RXFP1-BP. Data are mean ± SEM from 2 independent experiments. The competition binding data were fitted using a one site binding model.

The secondary structure propensity of the constrained H1 RLX B-chain analogues was explored by circular dichroism (CD) spectroscopy, performed both in phosphate buffer and SDS micelles (Figure 1), in comparison with the linear unmodified peptide [Ser^10^, Ser^22^] H1 RLX B (1–23) [**1**]. In general, CD spectra of the stapled peptides in phosphate solution (Figure 1A) showed a tendency to assume α-helical secondary structure, as compared to the linear reference Peptide **1** (green). Interestingly, the 3 peptides of the R series, characterized by a C-terminal oriented triazole stapling (**2**: pale green, **4**: blue, and **6**: light blue) are slightly more helical than the corresponding analogues, characterized by N-terminal oriented triazole (**3**: pale blue, **5**: brown, and **7**: light blue), as indicated by the more intense minimum at 222 nm. We subsequently performed CD spectroscopy in SDS micelles to explore the amphipathic properties of the analogues (Figure 2B), since the main aim of our design was to expose the key residues of H1 RLX B chain receptor binding cassette on the same face of a helical structure. Interestingly, all peptides showed a pronounced helicity in SDS micelles, including the linear reference **1** (green), although slightly less than the stapled ones. Among the stapled peptides, **2** (pale green) and **3** (pale blue) showed the highest degree of helicity (Figure 1B).

### Receptor binding assay

Measurements of specific binding to RXFP1-BP expressing HEK-293T cells of serelaxin, B7-33 and the H1 peptides under study are shown in Figure 2. Among the tested H1 peptides, only **6** and **7** and, at a lesser degree **4** demonstrated any ability to compete with Eu-H2 relaxin, albeit only with ∼20% competition of Eu-H2 relaxin binding at 10 µM.

### Signaling pathways downstream RXFP1 activation

**FIGURE 3:**
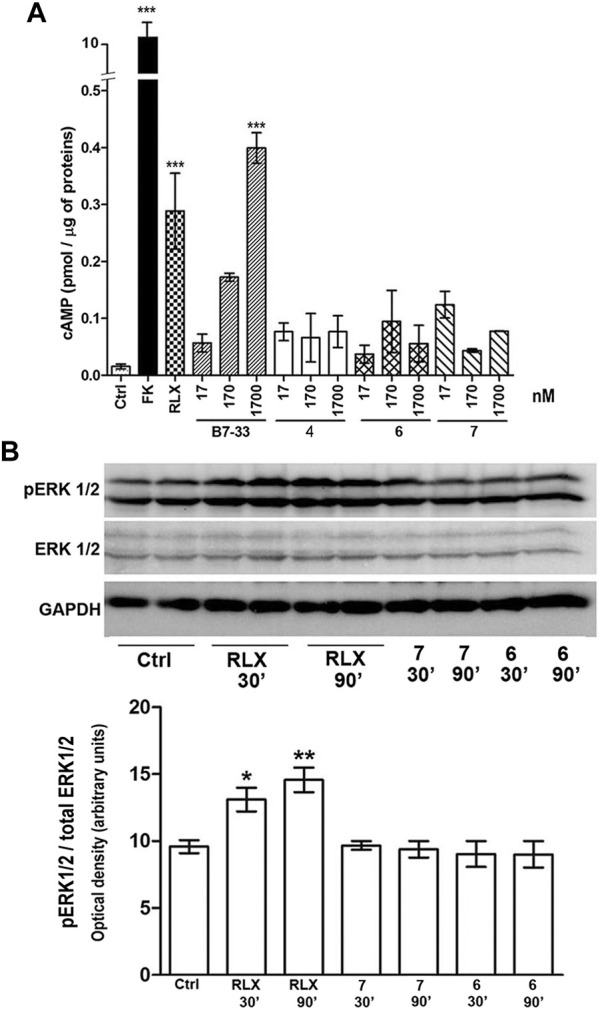
Assessment of RXFP1 signaling pathway activation. **(A)** cAMP generation in human monocytic THP-1 cells: both serelaxin (RLX) and peptide B7-33 induce a statistically significant elevation of cAMP, while none of the tested H1 peptides yielded a significant elevation of cAMP at any tested concentration. The adenylate cyclase activator forskolin (FK) was used to assess maximal cAMP yield. **(B)** ERK1/2 phosphorylation in mouse myofibroblasts: serelaxin induced a statistically significant increase in pERK1/2 at both 30 and 90 min, while none of the tested H1 peptides did. Values are mean ± SEM of 3 independent experiments. Significance of differences: ∗ *p* < 0.05, ∗∗*p* < 0.01, ∗∗∗*p* < 0.001.

Based on the results of the RXFP1 binding assay, H1 peptides **4**, **6** and **7** were selected for evaluation of possible RLX-like biological activity. This was assessed by measuring two main downstream signaling pathways, cAMP generation and ERK1/2 phosphorylation, on RXFP1-expressing human monocytic THP-1 cells and mouse TGF-β-induced myofibroblasts, respectively (Figure 3). In THP-1 cells, both serelaxin and peptide B7-33 induce a statistically significant elevation of cAMP, albeit B7-33 reaches values in the range of those achieved by RLX at a 100-fold higher molar concentration. Conversely, none of the tested H1 peptides yielded a significant elevation of cAMP at any tested concentration (Figure 3A). Similar results were observed in mouse myofibroblasts, obtained from NIH-3T3 fibroblasts stimulated with TGF-β for 48 h and identified by typical α-sma expression (Figure 3B). In these cells, serelaxin induced a statistically significant increase in ERK1/2 phosphorylation at both the tested exposure times (30 and 90 min), whereas neither of the H1 peptides **6** and **7** were capable of reproducing the effects of the authentic hormone, despite being added at 100-fold higher concentrations than serelaxin (Figure 3C).

## Discussion

Despite peptide drugs holding great promise of efficacy because they can mimic their natural bioactive counterparts, issues related to the oral route of administration are a major limitation to their use, since they are rapidly hydrolyzed and inactivated by the potent proteolytic enzymes of the stomach and small bowel. These issues can be partly addressed by enzyme-protected pharmaceutical formulations, which can withstand gastrointestinal digestion and release drugs in the distal small bowel, where these can be absorbed to the bloodstream chiefly through permeable lymphatic Peyer’s patches. In this context, demonstration has been provided that properly formulated insulin, which shares many structural similarities with RLX, can reach the circulation in therapeutically effective amounts upon oral delivery (Heinemann & Jacques 2009; Antunes et al., 2011), with obvious advantages in terms of patients’ compliance to the therapy. In spite of this caution, however, orally delivered peptide drugs still undergo partial digestion, which reduces the administered dose to unpredictable levels: in some instances, this limitation is counterbalanced by the fact that some proteolytic fragments may maintain, at least in part, the bioactive properties of the intact peptide. In the case of RLX, this possibility is substantiated by the observation that RLX analogues with truncations at the chain termini retain potent biological activity (Hossain et al., 2011).

In the present study we exploited a computational modelling chemistry approach, which allows to predict the molecular shape of a peptide based on its primary structure and its possible interactions with specific receptors, to design and synthesize a set of low molecular weight peptides on the putative receptor-binding domain of the H1 RLX B chain. Some of them were structurally stabilized in the appropriate 3-D conformation by intra-chain tri-azole staples, which should theoretically enhance their resistance to proteolytic enzymes (D’Ercole et al., 2020). Despite the favourable premises, none of the tested H1 peptides, either linear or stapled, revealed a substantial affinity to RXFP1 nor displayed any RLX-like biological effects, in terms of significantly relevant cAMP elevation and ERK1/2 phosphorylation in RXFP1-expressing cells. This was at variance with authentic serelaxin, which induced a clear-cut, significant activation of both the classical signaling pathways downstream RXFP1. Moreover, B7-33, designed on the receptor-binding domain of H2 RLX, was also able to induce a significant, although less prominent, cAMP response in the target THP-1 cells. The observation that this single-chain, linear analogue of H2 RLX maintains some efficacy in activating RXFP1, while the H1 RLX single-chain peptides described herein are not effective, suggests that differences between H1 and H2 RLX B-chain sequences, particularly in the N-terminal portion distal to the common receptor binding cassette, play a crucial role in receptor activation. In addition, we hypothesize that the triazole conformational constraint, devised to stabilize the bioactive conformation, plays on the contrary a negative effect, which does not favor the correct ligand-receptor interaction.

Albeit negative, the results of this study offer additional information about the structural requirements that new peptide therapeutics shall possess to effectively behave as RXFP1 agonists and RLX analogues. A more detailed conformational analysis, based on nuclear magnetic resonance (NMR) and molecular modelling, will enable the refinement of the design of the constrained analogues, changing the position and/or the extension of the triazole bridge, potentially leading to a better replica of the native bioactive conformation.

## Data Availability

The raw data supporting the conclusions of this article will be made available by the authors, without undue reservation.
